# Insight to Gene Expression From Promoter Libraries With the Machine Learning Workflow Exp2Ipynb

**DOI:** 10.3389/fbinf.2021.747428

**Published:** 2021-10-14

**Authors:** Ulf W. Liebal, Sebastian Köbbing, Linus Netze, Artur M. Schweidtmann, Alexander Mitsos, Lars M. Blank

**Affiliations:** ^1^ iAMB-Institute of Applied Microbiology, ABBT, RWTH Aachen University, Aachen, Germany; ^2^ AVT-Process Systems Engineering, RWTH Aachen University, Aachen, Germany; ^3^ Department of Chemical Engineering, Delft University of Technology, Delft, Netherlands

**Keywords:** machine learning, gene expression, strain engineering, biotechnology, synthetic biology, jupyter notebook

## Abstract

Metabolic engineering relies on modifying gene expression to regulate protein concentrations and reaction activities. The gene expression is controlled by the promoter sequence, and sequence libraries are used to scan expression activities and to identify correlations between sequence and activity. We introduce a computational workflow called *Exp2Ipynb* to analyze promoter libraries maximizing information retrieval and promoter design with desired activity. We applied *Exp2Ipynb* to seven prokaryotic expression libraries to identify optimal experimental design principles. The workflow is open source, available as Jupyter Notebooks and covers the steps to 1) generate a statistical overview to sequence and activity, 2) train machine-learning algorithms, such as random forest, gradient boosting trees and support vector machines, for prediction and extraction of feature importance, 3) evaluate the performance of the estimator, and 4) to design new sequences with a desired activity using numerical optimization. The workflow can perform regression or classification on multiple promoter libraries, across species or reporter proteins. The most accurate predictions in the sample libraries were achieved when the promoters in the library were recognized by a single sigma factor and a unique reporter system. The prediction confidence mostly depends on sample size and sequence diversity, and we present a relationship to estimate their respective effects. The workflow can be adapted to process sequence libraries from other expression-related problems and increase insight to the growing application of high-throughput experiments, providing support for efficient strain engineering.

## 1 Introduction

Metabolic engineering aims at optimizing metabolite production by adjusting the activity of native and heterologous enzymes. A frequently manipulated factor of activity is the enzyme concentration, which can be regulated on transcriptional, translational, and post-translational levels. In bacteria, enzyme concentrations are mainly set at the transcriptional level (Balakrishnan et al., 2021). Among the most important transcriptional element is the promoter sequence. The promoter sequence is the primary target for metabolic engineering because the expression activity is largely controlled by the sequence and can be easily altered. Both the composition and regulation of expression by promoters have been intensively studied (Kalisky et al., 2007; Zaslaver et al., 2009; Kochanowski et al., 2017). For example, Rhodius and coworkers investigated the expression strength of 60 *σ* ^E^ promoters and analyzed the impact of promoter boxes and upstream regulating elements on expression (Rhodius and Mutalik, 2010; Rhodius et al., 2012). A modular promoter system was developed by Mutalik et al. (2013), that reduces interference from the sequence of the gene of interest, resulting in reproducible promoter activities.

Promoter libraries are routinely constructed to generate promoters with a wide range of activities (Jensen et al., 1993; Alper et al., 2005; Hammer et al., 2006; Balzer et al., 2013; Köbbing et al., 2020) and machine learning has been applied for better understanding of transcription mechanisms and activity prediction based on sequence. For example, Meng *et al.* analyzed 98 *σ* ^70^ promoter sequences in *E. coli* and fine-tuned heterologous expression with newly designed synthetic promoters (Meng et al., 2013, 2017). The machine learning analysis therein was based on artificial neural networks (ANN) (Meng et al., 2013) and support vector machines (SVM) (Meng et al., 2017), and both approaches performed comparably. The same system with *σ* ^70^ driven expression in *E. coli* was tested by Zhao *et al.* with over 3,500 promoter sequences (Zhao et al., 2020) and analyzed using projection to latent spaces (PLS), tree methods (gradient boosting trees, GBT), and recurrent neural networks, wherein GBT performed best. In *Bacillus subtilis* Liu *et al.* employed a synthetic promoter library with 214 sequences to adjust pathway activity for metabolite overproduction (Liu et al., 2018) and used PLS for regression analysis. A promoter library with 80 sequences in *Geobacillus thermoglucosidasius* was tested for both the expression of GFP and mOrange and trained on models of PLS and ANN (Gilman et al., 2019). Overall, the libraries were generated with a varying sample sizes from 60 to over 3,500 and enabled model-based sequence analysis and rational promoter development.

Promoter libraries are typically analyzed by individually developed scripts, a time consuming process which impedes direct comparison of performance measures. To unify the analysis and provide a platform suitable for easy reconfiguration, we developed a general workflow for promoter library analysis called *Exp2Ipynb*. The *Exp2Ipynb* workflow consists of a collection of Python Jupyter Notebooks (RRID:SCR_018413) for statistical investigations, machine-learning, estimator performance evaluation, and sequence design by optimization of the estimator. We tested the workflow on an existing promoter library for *σ* ^70^ in *Pseudomonas putida* KT2440, expanded with new sequences. The results revealed limitations of one-factor-at-a-time experimental designs for machine learning. Subsequently, we used six published libraries to compare their composition and the resulting machine learning performance.

## 2 Methods

### 2.1 Data Preparation and Statistical Analysis

A configuration file stores variables with global use in each Notebook-assisted analysis. The data input is a comma-separated-value file (*csv*) with at least three columns: 1) an identifier column, 2) a sequence column, and 3) an expression value column, with header names. Optional columns are expression values of the sequences in other organisms or with a different reporter system and the standard deviation with the replicate numbers. The sequence column only accepts DNA abbreviations (A, C, G, T) with an identical length for each sequence. The output file names and figure file types can be defined. The column names for data import are defined in a separate configuration file *config.txt* and the notebook *0-Workflow* guides through its construction.

The statistical analysis in the Notebook *1-Statistical-Analysis.ipynb* provides an overview of the metrics with relevance for data exploration and model development. In addition to a single expression value, the standard deviation and replicate number can be provided. Optionally, outliers in the original data set can be removed from further analysis. Machine learning performance is improved if replicates are available and the workflow enables the re-generation of replicates based on mean and standard deviation. The replicates are calculated from Python *numpy* (RRID:SCR_008633) random normal function (Harris et al., 2020) and are valid for normal-distributed data while adding a reasonable prediction bias.

**FIGURE 1:**
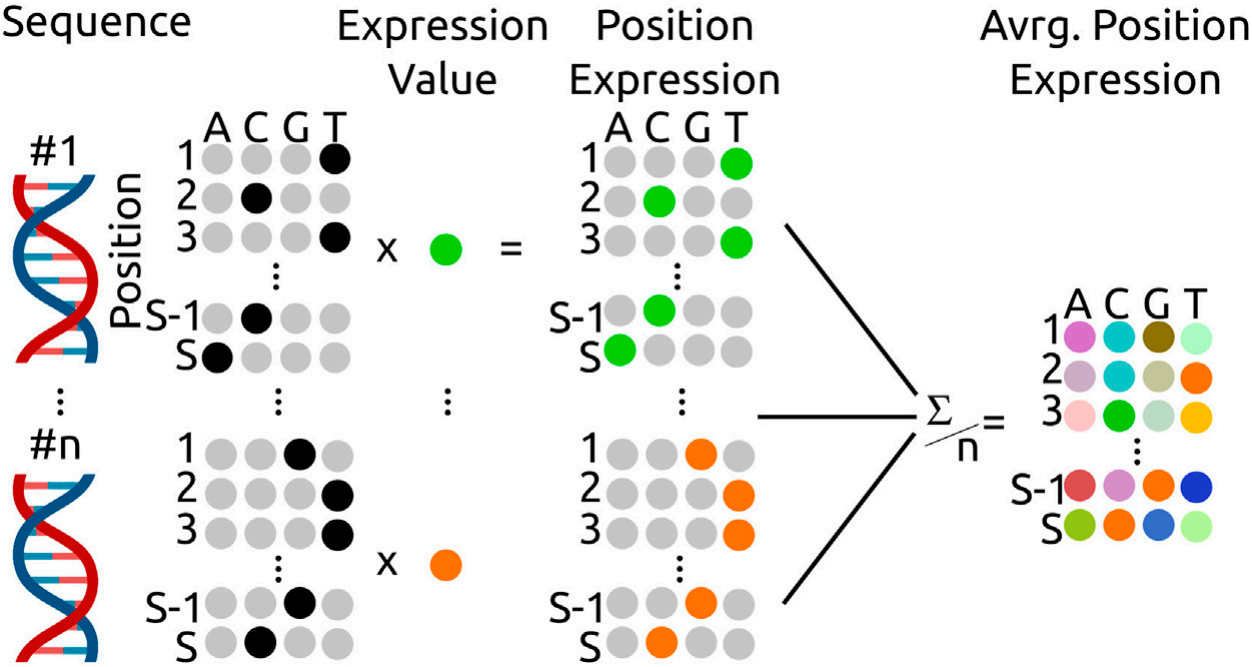
Calculation scheme for the average, position dependent expression $\bar{Exp}$ . The one-hot encoding of each promoter sequence is multiplied by the sequence dependent expression strength to yield the position expression. The position expressions are summed and divided by the sample number to arrive at the average expression for each nucleotide at each position.

The sequence diversity represents how different the sequences are from each other. It is calculated as the normalized sum of nucleotide differences relative to a reference sequence or among all sequences and ranges between 0 (identical) to 1 (each position differs). The reference sequence can be provided with the configuration file, or it is generated automatically by finding the most common nucleotide on each position. For large libraries, i.e., >1000 samples, the total pairwise distance is costly to compute and the reference sequence distance is performed by default. The position diversity informs about how many nucleotides have been sampled for each position. It is visualized with two bar plots: 1) the cumulative number of each nucleotide tested on each position, and 2) the entropy (*H*_*i*_) for any sequence position (*i*):

$$H_{i}=-\underset{i=\left(A,C,G,T\right)}{\sum }p_{i}\log_{2}p_{i}$$ (1)

where *p*_*i*_ is the position-related nucleotide frequency. The expression statistics for all nucleotides and positions $\bar{Exp}$ informs how each nucleotide contributes to the expression and its calculation is shown shematically in Figure 1. Each sequence is transformed into a one-hot encoding with the four nucleotides as columns (A, C, G, T) and the sequence as rows with the maximum sequence length *R*. This sample-sequence one-hot matrix is multiplied by the associated expression strength (*Exp*_*n*_) and is used to calculate the mean (standard deviation) of expression at each position-nucleotide pair over all samples (*S*). The expression strength is visualized with a histogram and a scatter plot for cross-library expression.

### 2.2 Machine Learning Training

The workflow supports classification and regression. In regression, the expression values for sequences are quantitatively predicted but require high data quality (sufficient sample size and position entropy). Classification provides qualitative predictions (e.g., low-medium-high), with more reliable predictions even for small data sets. The implemented machine learning models are random forest (RF), gradient boosting trees (GB), and support vector machines (SVM). The input features are nucleotides on each position in one-hot encoding plus the overall sequence GC-content. The predicted target variable is the expression strength. The feature size depends on the sequence length, typically ranging between 40–100 nucleotides or 160–400 features. If a classification is chosen, the output is binned according to a parameter provided by the user (*Response*_*Value* in *config.txt*). If *Response*_*Value* = 1, the original activity values are used; if the *Response*_*Value* = 0, the data is centered with zero mean and unit variance and for larger values, bins are generated as equal-sized buckets (python *pandas qcut*, RRID:SCR_018214), and the bin label is used as the target prediction. The data is split into training and test set, with a default ratio of 9:1 and a grid search on the training set identifies the optimal hyper-parameter with 100 fold cross-validation.

Additional feature selection procedures were implemented to increase performance. During feature selection, the number of nucleotides that serve as features can be reduced to optimize training. Reasonable predictions rely on a sufficient variety of nucleotides tested at each position (*H*_*i*_), and a cut-off can be chosen in the configuration file to exclude positions below a defined value of *H*_*i*_. However, note that unconserved nucleotides in conserved promoter (box)-regions can result in severe expression deficiencies. Thus, a low diversity can also reflect critical nucleotides associated with difficulties to sample experimentally.

### 2.3 Machine Learning Performance

The performance evaluation provides metrics for correlating the experimental and predicted outcomes and informs about important features for tree-based methods. The performance evaluation is based on 25-fold cross-validation with 9:1 data separation that jointly moves identical sequences (i.e., replicates) to test- or training- sets. Thus, the test sequences are unknown to the regressor. The performance evaluation is based on the R2- (regression) and weighted F1-score (classification) (and optionally Matthews correlation coefficient) for the training set and the test set. The prediction uncertainty metric is determined by the coefficient of variance of the mean R2 and F1 prediction of the cross-validated training set. The tree-based methods allow the extraction of feature importance and represent the contributions of each nucleotide-position and are visualized with Logo-plots (Tareen and Kinney, 2020). Moreover, single decision trees can be exported.

### 2.4 Promoter Design by Optimization

The ability to design new promoters with desired activities is required for effective strain engineering. The predictors resulting from the machine-learning training are used to search promoters and expression with a genetic algorithm based on the Python framework DEAP (Fortin et al., 2012). A genetic optimization algorithm is used as it can be easily applied to a wide variety of machine learning models. The initial population is composed of random sequences and point mutations and crossing-over is used to search the sequence space. Sequence identification for a regressor is conducted with a search for the sequence with the closest expression. The classification requires that the predicted expression lies within the target expression class while the sequence distance to the reference sequences is minimized. The sequences already present in the library are excluded from the search.

### 2.5 Experimental Library Construction

The following section describes the experiments conducted to expand an existing promoter library (Köbbing et al., 2020) which was used to test the machine learning and experiments to test newly designed promoters from the associated estimator optimization. Construction of the single nucleotide polymorphism (SNP) library was based on the plasmid pBG14g as template with oligonucleotides containing single degenerate nucleotides inside the P14g promoter sequence (Zobel et al., 2015; Köbbing et al., 2020). The fragments and vector pBG were digested with *Pac*I and *Nco*I (New England Biolabs) at 37°C. Digested backbone and promoter containing PCR fragments were ligated with T4 ligase (New England Biolabs) at room temperature for 30 min. Transformation into chemically competent *E. coli* PIR2 cells was done by heat shock (Hanahan, 1983). Plasmids containing different synthetic promoter sequences were sequenced (Eurofins Genomics) and genomically integrated into the *att*Tn7 site of *P. putida* KT2440 by mating (Zobel et al., 2015). For promoter characterization in *P. putida* KT2440, cells were grown in minimal medium (Hartmans et al., 1989), with 20 mM glucose as carbon source. The Biolector system (M2P Labs) measured optical density (620 nm) and msfGFP (excitation wavelength 488 nm, emission wavelength 520 nm). Scattered light was correlated to OD600 with a dilution series of a stationary phase culture. Promoter activity reflects the slope of the function of fluorescence over OD600 at the beginning of the exponential phase. More detailed information is given in Köbbing et al. (2020).

## 3 Results

### 3.1 Case Studies

The workflow was first applied on a newly extended *P. putida* KT2440 synthetic promoter library, followed by a cross-analysis of six published libraries. The study of our *P. putida* library will show how the workflow can be used, and we will highlight shortcomings of the experimental design for machine-learning-driven research. In the cross-library comparison, we investigated how the different library parameters of sample size, diversity and feature number of the input sequences impact the prediction quality of expression strength.

### 3.2 *P. putida* Single Library Analysis

**FIGURE 2:**
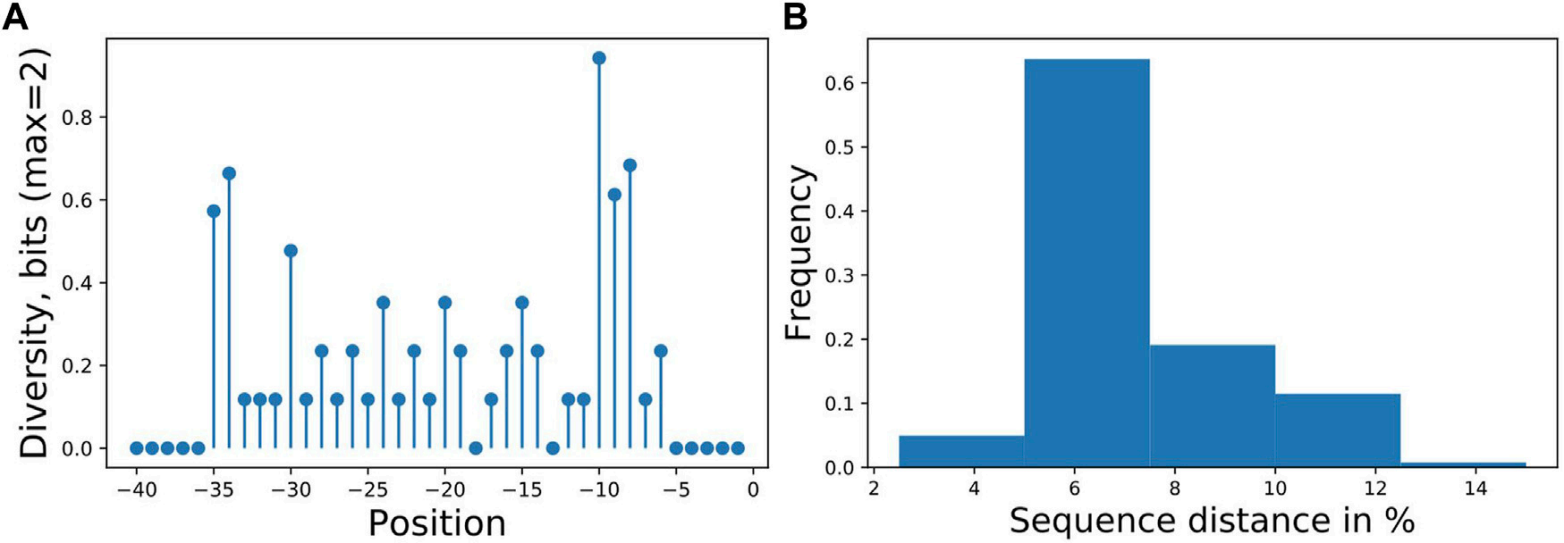
**(A)** Promoter sampling diversity of the complete data set for nucleotide variations on each sequence position, transcription starts at 0 and **(B)** mutual sequence distances. The promoter library is based on our previously published library (Köbbing et al., 2020), with additional sequences totaling 63 different promoters with mutations directly upstream of the transcription start site. Two positions (−13,−18) were not mutated resulting in 28 tested positions.

We used the workflow to analyze a synthetic library in *P. putida* KT2440 driven by *σ* ^70^-dependent promoters and measured with GFP published previously (Köbbing et al., 2020) containing 55 unique promoter sequences, here expanded by eight new sequences with an overall sample size of the library of 63. The goal was to identify sequence features responsible for expression strength. Experimentally, the sequence diversity was generated by single nucleotide exchanges in 28 nucleotides upstream of the transcription start site. For the analysis, we used 40 nucleotides of the promoter as input and binned the expression activity into three approximately equal classes for the output. The sequences had low information content on most positions (Figures 2A,B), allowing only predictions of categorized expression values, a regression predicted not better than random (not shown).

**FIGURE 3:**
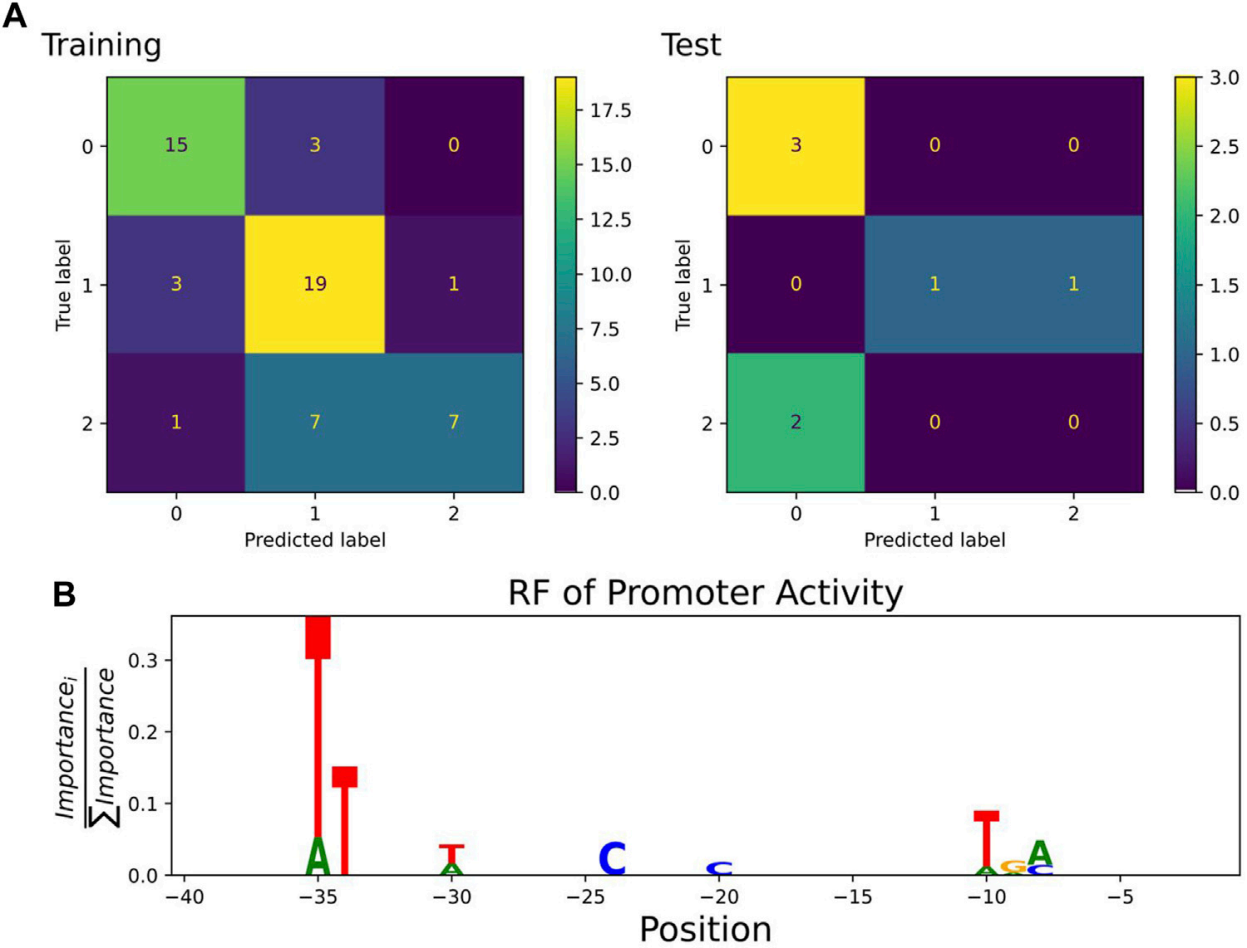
**(A)** Confusion matrix showing classification quality for training (*n* = 56) and test set (*n* = 7) as predicted by the best random forest estimator. The classification labels are 0: low (expression 0.2–16.6), 1: medium (16.6–24.8), 2: high (24.8–35.2) (Köbbing et al., 2020). **(B)** Sequence logo of positions used by the estimator to predict expression activity class. The −35 and −10 box positions dominated the feature importance.

A classification estimator can predict the approximate magnitude of expression. A reasonable estimation can only be performed on features with sufficient information content and positions with a higher entropy than 0.2 bits were included in the training. Figure 2A shows the entropy of the complete data set, and because the cut-off is applied to the training subset, the following positions are additionally neglected (−6,−15,−20,−22,−26). Decreasing the entropy threshold did not affect the performance (not shown). The classification details are given in Table 1, and the high F1-score variation indicates that the low nucleotide redundancy on each position affected sample separation during cross-validation. The detailed prediction results of the training and test set are shown in Figure 3A. The GC-content is among the most important features, probably because it has the highest entropy of all features (1.2 bits). Along with the GC-content, critical features were the positions –35 and –34, corresponding to the fact that the -35-box is the site of transcription factor binding (Paget, 2015) (Figure 3B). New sequences with defined expression activity were identified. Sequences with close relation to the reference sequence were chosen, because the data used for training itself has a low sequence diversity (Figure 2). Three promoters were suggested by the optimization procedure, two with low expression and one with high expression and were experimentally tested (see Table 2). While the newly designed promoters with low activity could be validated, the designed promoter with a predicted high activity showed a medium activity experimentally.

**TABLE 1:** Classification quality report for random forest (RF), gradient boosting trees (GBT) and support vector machine (SVM). *Run time* reflects the computational time to train the respective machine-learning algorithm. *CV:F1-score* is the result of cross-validation performed 25 times with split 9:1 on the training data. *Train/Test:F1-score*, *GC-content*, and *Top 3 FI* are results of the best respective estimator for training and test set F1-score, the importance of the GC-content feature and the thre most important sequence positions along with the importance values. The training was performed with the same Train(56)-Test(7) division with cross-validation on the training set (100 times 9:1 split). Nucleotides included as features were filtered to contain at least an entropy of 0.2 bits, which resulted in 15 positions, in addition to GC-content (input vector: 15 × 4 + 1). Only tree-based methods (RF, GBT) extract the feature importance (FI).

	RF	GBT	SVM
Run time (s)	144	927	111
CV:F1-score	0.42 ± 0.19	0.5 ± 0.22	0.47 ± 0.21
Train:F1-score	0.58	0.89	0.88
Test:F1-score	0.62	0.46	0.14
GC-content	2nd	1st	N.A.
Top 3 FI	−35: *T*: 0.24	−34: *T*: 0.08	N.A.
	−34: *T*: 0.10	−35: *A*: 0.05	N.A.
	−35: *A*: 0.10	−14: *G*: 0.05	N.A.

**TABLE 2:** Performance of newly designed promoters. The reference promoter *Ref:mod4_1* was measured in the original data set and differs from the designed promoters only by single nucleotide changes. In the original data set, the reference promoter displayed a GFP expression activity of 9 ± 2 Units.

ID	Seq.Diff	Prediction	Experiment
Ref:mod4_1	GGTATAAT	0.25 − 16.5	6.5 ± 0.3
pred:0–1	GGTA**C**AAT	0.25 − 16.5	7.1 ± 0.9
pred:0–2	GGTAT**T**AT	0.25 − 16.5	9.7 ± 2.5
pred:2–1	**C**GTATAAT	25 − 35	18.3 ± 1.7

### 3.3 Cross-Library Analysis

In the following, six published bacterial promoter libraries were analyzed separately to highlight the effect of the library design on the estimation quality. The data is available online in the *Exp2Ipynb* package at GitHub. The libraries were measured in *E. coli*, *Geobacillus thermoglucosidasius*, and *B. subtilis* and were targeted to specific or unknown sigma factors. All libraries differ in terms of combinations of library sample size, tested sequence length and sequence diversity (Table 3).

**TABLE 3:** Details of the six published libraries used to compare estimation quality in response to the experimental design. The columns *n* represent total sample size, *Feat.* the size of the input vector, *Avg.Seq.Distance* the average distance of all sequences to a reference sequence, composed of the most common nucleotide at each position. A comprehensive table with numerical values of Figure 4 in the Supplementary Data. Multiple transcription factors are responsible for gene expression in the data set of Gilman et al. (2019), hence is not applicable (*N.A.*).

Reference	Organism	Transcr.Factor	Reporter	*n*	Feat	Avg.Seq.Distance
a:This Work	*P. putida*	*σ* ^70^	GFP	63	61	0.06
b:Rhodius *et al.*	*E. coli*	*σ* ^E^	various	59	121	0.61
c:Meng *et al.*	*E. coli*	*σ* ^70^	GFP	98	709	0.61
d:Zhao *et al.*	*E. coli*	*σ* ^E^	GFP	3543	285	0.09
e:Gilman *et al.*	*G. therm.*	N.A.	GFP	81	397	0.69
f:Gilman *et al.*	*G. therm.*	N.A.	mOrange	81	397	0.69
g:Liu *et al.*	*B. subtilis*	*σ* ^A^	GFP	206	105	0.35
h:Meng *et al.*	*E. coli*	*σ* ^70^	GFP	84	133	0.61
i:Meng *et al.*	*E. coli*	*σ* ^70^	GFP	84	129	0.61
k:Zhao *et al.*	*E. coli*	*σ* ^E^	GFP	896	161	0.09

The analysis was based on a classification task with a random forest and three classes: low (*1*), medium (*2*), and high expression (*3*). The original data of the promoter libraries were used with minor corrections regarding sequence length homogenization. For quality assessment, we generated three synthetic data sets based on partitioned sequence regions from Meng et al. (2013) and Zhao et al. (2020). The original sequence from Meng et al. (2013), is 224 nucleotides (nt) long, and we extracted the starting 40 nt (*h*) and last 40 nt (*i*), assuming that few positions in the new sequences influence expression. Zhao et al. (2020), tested 113 nt ranging from upstream regulating elements down to the coding sequence. Again, the first 40 nt (*k*) were extracted, corresponding to the upstream regulating element. Three values are essential for the analysis of promoter libraries: 1) the fidelity of gene expression prediction, computed by the F1-score (see methods), 2) the confidence of the prediction, computed by the coefficient of variation, and 3) critical sequence features for expression, computed by the feature importance of random forest and gradient boosting strategies.

**FIGURE 4:**
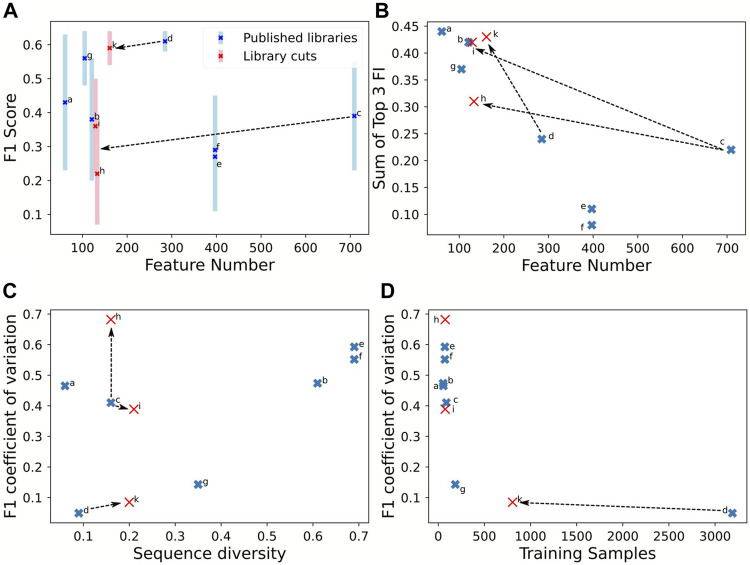
Comparison of library properties on classification quality parameters for an estimation using RF. The estimations were performed on the training set of each library with 9:1 cross-validation. The seven full length promoter libraries are listed in Table 3. Moreover, sub-sequences of 40 nt were extracted from the promoter start (*h*) and end (*i*) of Meng et al. (2013)(*c*) and start (*k*) of Zhao et al. (2020)(*d*). F1-score **(A)** and the sum of the top three feature importance values **(B)** in response to feature amount. The coefficient of variation of the F1-scores in response to sequence diversity **(C)** and amount of training samples **(D)**. Full table with numerical values in the Supplementary Data.

The prediction qualities, evaluated by the F1-score, ranged between 0.3 and 0.6. The promoter library with more than 3,500 samples achieved best predictive quality (F1-score) and confidence (coefficient of variation of F1-score) (Figure 4A, library *d*). Higher sample size was associated with lower sequence diversity and consequently better prediction confidence, with coefficient of variations below 0.2 (*d*, *g*). Notably, the F1-score was independent of the number of tested input features (Figure 4A). The effect of the training sample size on the coefficient of variation is hyperbolic decreasing (Figure 4C): below 200 samples represented by library *g*, the coefficient of variation rises, thus decreasing prediction confidence. Two scenarios are visible in Figure 4C, *a* and *c* display already a very low sequence diversity and for improving performance the sequence diversity should be increased. In contrast, libraries *b*, *e*, and *f* are diverse, and estimation performance would benefit by increasing the sample size even with related sequences. An interesting piece of information is the feature importance, for features that correlate with expression activity. Figure 4B indicates how the number of features affects the sum of the three top important features derived from the RF. The top-three feature importance sum decreases linearly with increasing numbers of features. However, for library *c*, the top three nucleotide positions are much more predictive than expected, assuming that more features lead to a larger distribution of feature importances and thus less proportional share for the top three features available.

Using sequence subsets to test the effect of the number of features confirmed lower importance of feature numbers compared to sequence diversity and sample size. To test sequence subsets, we chose the libraries from Meng et al. (2013) and Zhao et al. (2020) because they report a high feature number with low sequence diversity. Sub-sequences from Meng et al. (2013) were extracted with 40 nt sequences at the start (*h*) and end (*i*) because these contain features with low importance. The rationale to use the starting 40 nt from Zhao et al. (2020) (*k*) is that they included the upstream regulating element and are essential expression features. The sequence extraction reduces the sample size because more redundant sequences are generated, increasing the nucleotide diversity. Figure 3 shows that the partitioned data set *k* (Zhao et al., 2020) maintained the prediction confidence from the original data set: the F1-CoV and top three feature contribution increased proportionally to the original data (Figures 4B–D). This proportional movement was not displayed by the extracted sequences (*h*, *i*) from Meng et al. (2013). Thus, sequence positions impacting expression were identified in the upstream regulating elements of Zhao et al. (2020), but not in the first and last 40 nt of the sequences of Meng et al. (2013). The synthetic data set *k* is in line with the sample size correlation to the coefficient of variance of the libraries *d* and *g*. These results indicate that sample size in the range of 200 to 3,500 is not critical for the coefficient of variance.

## 4 Discussion

Synthetic promoter libraries are increasingly constructed to facilitate promoter selection with defined activities. The *Exp2Ipynb* workflow supports the analysis of promoter libraries to identify the sequence information that determines expression strength. The identification is based on statistical analysis of the average nucleotide-position associated expression, and the training of different machine-learning models. Moreover, more complex libraries with multiple readouts, like two reporter proteins, transcript, protein level, or cross-host expression can be analyzed. The workflow facilitates data exploration, regressor training and performance evaluation, and testing of novel sequences within the DNA sequence exploration space. The implementation in a Jupyter notebook facilitates rapid implementation and the low-level scripting allows for direct adaptation of the workflow to specific needs. In the following, we summarize the applicability of *Exp2Ipynb* to a *P. putida* promoter library with sub-optimal data quality, followed by a discussion of data properties for optimal sequence analysis.

We analyzed a promoter expression library in *P. putida* KT2440 previously published (Köbbing et al., 2020) and amended it with additional data points and used the classification model to design new promoters with defined activity. The different samples in the library were generated based on a one-factor-at-a-time approach to identify nucleotide-sequence effects on expression (Czitrom, 1999). Our results of the strong impact of the positions −35 and −34 and the lower impact of the last half of the −10 box (TATAAT, −10, −9, −8) confirmed results of the original article (Köbbing et al., 2020, Figure 4 therein). However, we failed to observe the strong effect of the first part of the −10 box (TATAAT, −11) because the position was insufficiently sampled below the entropy cut-off and was neglected in the analysis. We used the classifier to apply a genetic algorithm and find sequences within a defined expression range and we confirmed their expression strength experimentally. Thus, even data sets with low sample size and sequence diversity allow for predicting expression ranges for biotechnological use.

Sample size and sequence diversity were the main factors to affect prediction confidence (coefficient of variation) while the number of feature was less critical. A high sequence diversity resulted in lower prediction confidence, and increasing the sample size can help to reduce diversity and increase prediction confidence. Two libraries in the collection were limited by sample size (*a*+*c*), whereas five libraries were limited by sequence diversity (*b*, *d*, *e*, *f*). We found that 100 samples still resulted in a high uncertainty for sequences with an average of 20*%* nucleotide difference (*a*, *c*), whereas 800 samples were sufficient (*k*). With higher nucleotide differences of 35*%*, 200 samples provided reasonable prediction qualities (*g*). Libraries including multiple sigma factors (*e*, *f*)(Gilman et al., 2019) resulted in lower prediction qualities, which parallels studies on heterogenous data in *E. coli* (Cambray et al., 2018) and yeast (Liya et al., 2021). More libraries are necessary to narrow the required sample size over the whole sequence diversity spectrum.

Our prediction quality (F1-score) performs poorly over the different libraries. Some studies have identified much stronger regression correlation coefficients (Rhodius and Mutalik, 2010; Meng et al., 2013, 2017). These were based on optimized train-test set partitions and do not report cross-validation statistics. Gilman *et al.* calculate a stronger correlation for their model, but when the authors tested new data, the prediction qualities were similar to those we observed. Also, the convolutional neural net trained with 500,000 sequences by Cuperus et al. (2017), achieves correlation coefficients of 0.62. Additional feature engineering can be used to increase predictability: GC-moving window, secondary structure for UTR (Cuperus et al., 2017). Multiple factors control gene expression of which a number is apparently not stored in the sequence alone.

The *Exp2Ipynb* workflow includes training of RF, GBT and SVM, whereas neural-networks are not explicitly implemented. In the original articles of the libraries tested here, SVM and GBT performed comparably or outperformed neural network based approaches (Meng et al., 2017; Zhao et al., 2020). Most promoter libraries have samples sizes on which neural networks are not expected to outperform classical methods. The performance of each machine learning model depends on the underlying data structure, and the most suitable method has to be identified individually (Wolpert and Macready, 1997). It is possible to include additional methods via the python interface easily.

So far, the analysis of promoter libraries was conducted with scripts tailored to the data, obfuscating reproducibility and interpretability. The *Exp2Ipynb* workflow contributes to harmonize analytical workflows and enables an easy start for investigating newly generated promoter libraries. Other general machine-learning toolboxes exist, e.g*.*, *tpot*, *GAMA*, or *H2O* (Truong et al., 2019), in addition to tools more oriented towards biological data analysis, e.g*.*, *JADBIO* (Tsamardinos et al., 2020). The advantage of the *Exp2Ipynb* is to be general enough to be used across different data sets in expression studies but remaining domain-specific to facilitate simple data integration. We present a workflow called *Exp2Ipynb* for machine-learning supported analysis of gene-expression libraries. We applied *Exp2Ipynb* in a proof-of-concept on a *P. putida* KT2440 promoter library for use in metabolic engineering. In this context, the workflow allowed identifying critical sequence features for expression and predicted new sequences with defined activity, tested retrospectively. Moreover, six published prokaryotic gene expression libraries were tested and we observed a correlation between sample size and sequence diversity for successful analysis. The workflow supports deep analysis of promoter libraries and allows users to adapt it to personal needs. Thus data quality assessments are improved and research is accelerated.

## Data Availability

Publicly available datasets were analyzed in this study. This data can be found here: https://github.com/iAMB-RWTH-Aachen/Exp2Ipynb.
